# α-Lipoic Acid Derivatives as Allosteric Modulators for Targeting AMPA-Type Glutamate Receptors’ Gating Modules

**DOI:** 10.3390/cells11223608

**Published:** 2022-11-15

**Authors:** Mohammad Qneibi, Safa’ Nassar, Sosana Bdir, Adel Hidmi

**Affiliations:** 1 Department of Biomedical Sciences, Faculty of Medicine and Health Sciences, An-Najah National University, Nablus P400, Palestine; 2 Department of Chemistry, Faculty of Sciences, Birzeit University, Birzeit P627, Palestine

**Keywords:** AMPA receptor, neuroprotective, α-Lipoic acid, desensitization, inhibition

## Abstract

The ionotropic glutamate receptor subtype α-amino-3-hydroxy-5-methyl-4-isoxazole propionic acid (AMPA) is responsible for most excitatory transmission in the brain. AMPA receptor function is altered in numerous neurological illnesses, making AMPA receptors appealing therapeutic targets for clinical intervention. Alpha-Lipoic acid (α-LA) is a naturally occurring compound, which functions as a co-factor in metabolism and energy production. α-LA is an antioxidant with various benefits in treating diabetes, including managing symptomatic diabetic neuropathy. This study will test a novel and innovative strategy to synthesize a new isomer of lipoic acid (R-LA) derivatives (bifunctional NO-donor/antioxidant) in one chemical on homomeric and heteromeric AMPA receptor subunits. We used patch-clamp electrophysiology to examine LA derivatives expressed in human embryonic kidney 293 cells (HEK293) for inhibition and changes in desensitization or deactivation rates. LA derivatives were shown to be potent antagonists of AMPA receptors, with an 8–11-fold reduction in AMPA receptor currents seen following the delivery of the compounds. Furthermore, the LA derivatives influenced the rates of desensitization and deactivation of AMPA receptors. Based on our results, especially given that α-LA is closely connected to the nervous system, we may better understand using AMPA receptors and innovative drugs to treat neurological diseases associated with excessive activation of AMPA receptors.

## 1. Introduction

Rapid excitatory transmission in the neural pathways of the central nervous system, as well as synaptic potentiation and depression, can be enabled by ionotropic glutamate receptors [1,2]. The α-amino-3-hydroxy-5-methyl-4-isoxazole propionic acid receptors (AMPARs), a subfamily of ionotropic glutamate receptors, play crucial roles in glutamatergic synapses in the hippocampus [3,4]. AMPARs are tetrameric assemblies of four subunits, GluA1–GluA4 [5], with GluA1 and GluA2 being the most abundant in the brain [6,7]. Each AMPA receptor subunit is composed of an extracellular amino-terminal domain (ATD), an extracellular ligand-binding domain (LBD), a transmembrane domain (TMD), and an intracellular carboxyl-terminal domain (CTD) [8,9]. AMPA receptors form dimers of dimers, with ATD interactions appearing to facilitate dimer formation. The LBDs and TMDs interact for tetramerization (forming two subunit dimers) [8].

Glutamate is the primary mediator of excitatory synaptic transmission in the brain and plays a vital role in critical brain activities [10]. Glutamate activates AMPA receptors by attaching to the cleft between the LBD domains D1 and D2. AMPA-type channels can bind up to four glutamate molecules since each AMPA receptor subunit can form functional channels. It is necessary to occupy one or two of the four subunits for activation gating, and the unitary conductance of AMPA-type channels rises with receptor occupancy [11,12]. The AMPAR’s extraordinary fast gating properties on the sub-millisecond timescale enable rapid depolarization of the postsynaptic membrane, permitting high-fidelity impulse transmission between nerve cells. In the presence of glutamate over an extended period, AMPA receptors desensitize within a few milliseconds. It was proposed that AMPA receptor desensitization necessitates a coordinated conformational shift of all four AMPA receptor subunits [11,13,14]. The glutamatergic system is vital in synaptic plasticity, learning, and memory under normal conditions, but it is also a robust neuronal excitotoxin in pathological conditions, causing either fast or delayed neurotoxicity [15,16,17]. As a result, careful control of glutamatergic neurotransmission is essential to maintain optimal neuronal function and prevent the system from becoming overactive.

**Figure 1:**
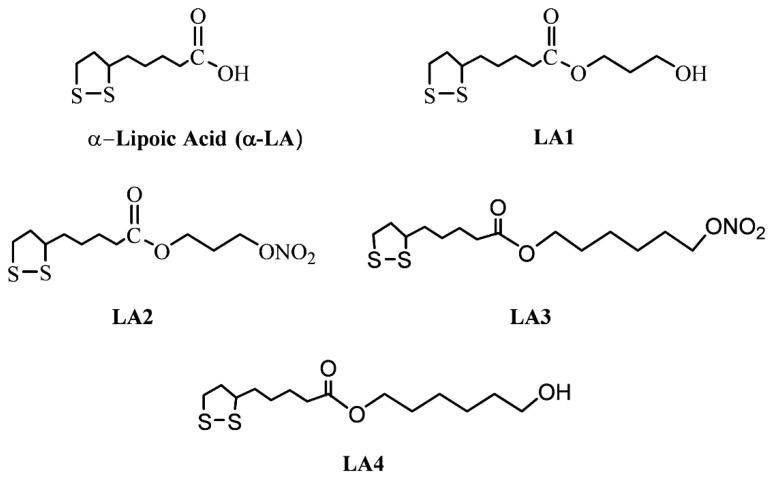
Chemical structure of α-Lipoic acid and its derivatives.

Several neurological and psychiatric diseases, including Alzheimer’s disease, Parkinson’s disease, Huntington’s disease, Amyotrophic Lateral Sclerosis, schizophrenia, obsessive-compulsive disorder, and mood disorders, have been linked to abnormalities in the regulation of extracellular glutamate concentrations [18,19]. Controlling AMPAR activity is being investigated as a potential treatment for neurological and psychiatric conditions. Even though there has been an increase in the development of AMPAR positive and negative allosteric modulators (PAMs and NAMs) [20], they have been found to lack selectivity for distinct brain areas [21,22,23]. For example, the NAM perampanel is effective against various seizure types, but its lack of regional specificity leads to side effects such as ataxia and dizziness [22,24]. These compounds bind to allosteric sites on AMPA receptors, slowing deactivation by keeping the clamshell in its closed-cleft, glutamate-bound conformation and improving signaling via the receptors [25]. Thus, our study presents four novel alpha-lipoic acids (α-LA) derivatives (Figure 1) to be examined on AMPA receptor subunits, as well as how AMPAR biophysical gating properties (i.e., deactivation and desensitization) might be affected. Furthermore, homomeric and heteromeric AMPA receptor subunits were evaluated since a balance of homomeric and heteromeric assembly methods allows for further AMPA receptor response adjustment under various physiological and pathological situations. Since more than 30 distinct constituents of AMPA receptor complexes have been identified, our research focused on AMPA receptor subunits rather than AMPA receptor complexes. The composition of these receptor complexes varies among brain regions, cell types, developmental stages, and, importantly, cellular compartments. The diverse composition of AMPAR complexes is explained by the varied roles of AMPAR interacting proteins. Future structural investigations are required to establish the interaction domains of AMPA receptor/auxiliary protein complexes and to comprehend the implications of these different functional connections [26,27].

In 1951, α-LA was a key component of energy metabolism [28,29]; it is an amphipathic molecule with both hydrophilic and hydrophobic characteristics [30]. α-LA, related to the genetic modulation of intrinsic antioxidant and anti-inflammatory pathways, may be synthesized in sufficient quantities by a healthy human body [31]. However, age and certain disorders can reduce the amount of α-LA in the body [32]. α-LA is found in plant sources such as spinach, broccoli, tomatoes, and animal tissue, with the most significant amounts in kidneys, heart, and liver containing 2.6, 1.5, and 0.9 × 10^−3^ g lipoyllysine/g dry weight, respectively [33,34]. Various cell lines were used to evaluate the effects of α-lipoic acid; one study examined the effect of α-lipoic acid on HEK293 cells and used 8 µM of α-lipoic acid to assess its antioxidant properties without noticing any harm [35]. On EA.hy926 endothelial cells, a dosage of 40–80 µM α-lipoic acid was applied without causing toxicity [36]. When used in appropriate doses, α-lipoic acid cannot be considered a cell toxin. In this study, each α-lipoic acid derivative will be administered starting from very low concentrations (2 µM), and the cells will be rinsed for 20 s between α-LA compounds, and a 10 mM glutamate will be administered alone after each α-LA compound to ensure cell health. Recently, there has been much focus on the efficacy of α-LA treatment in central nervous system (CNS) diseases. There are several reasons for this interest, the most noteworthy of which are the neuroprotective impact of α-LA on the CNS and its potential to pass the blood-brain barrier (BBB) [37]. Its antioxidant properties can influence the onset of several disorders, including multiple sclerosis and Parkinson’s disease. Many trials on the therapeutic use of α-LA revealed that α-LA administration could significantly relieve the symptoms of certain disorders or prevent their progression [30]. These properties combine to make α-LA a very promising supplement for the treatment of brain disorders and for modulating AMPA receptors.

## 2. Materials and Methods

### 2.1. Chemistry

Reagents, solvents, and other chemicals were purchased from (Sigma Aldrich-Merck Chemicals, St. Louis, MO 63103, USA), such as α-Lipoic acid (α-LA) 89%, 1,3-propanediol 98%, 1,6-hexanediol 97%, N,N′-Dicyclohexylcarbodiimide (DCC) 99%, sulfuric acid (H_2_SO_4_) 95.0–98.0%, nitric acid (HNO_3_) 99%, phosphoric acid (HNO_3_) > 85%, dichloromethane (DCM) ≥ 99.8%, methanol 99.8%, hexane 99.8%, 2-(1-Naphthylamino) ethylamine dihydrochloride 98%, napthaylamine diamine dihydrochloric acid >98%, ~85%, tetramethyl silane >99.9%, chloroform-d 99.8 atom% D, Dimethyl sulfoxide-d6 99.96 atom% D.

#### 2.1.1. Purification

The synthesized compounds were purified using flash column chromatography on Merck silica gel 60 (particle size 230–400 mesh), with the eluent combining CH_2_CL_2_ and CH_3_OH in various ratios. Analytical TLC was made on silica gel 60 F 254-percolated plates supplied by Merck, using UV light or I2 vaper to observe the purity of the compounds.

#### 2.1.2. NMR Measurements 

The VNMR software processed the data and captured it on a Varian VXR-300 (300 MHZ) spectrometer with a 5-mm switchable probe. The internal standard was tetramethyl silane Me_4_Si (0.00 ppm), and the solvents were chloroform-d and dimethyl sulfoxide-d6. The abbreviations for the splitting patterns are s, singlet; d, doublet; t, triplet; q, quartet; m, unresolved multiple due to instrument field strength; and dd, doublet of the doublet. ^1^H NMR and ^13^C NMR spectroscopic techniques were used to characterize and identify the synthesized compounds.

#### 2.1.3. Assessment Nitric Oxide Release

Nitric oxide (NO) was estimated according to the Greiss reagent kit (Promega Corporation, Madison, WI, USA). In brief, the concentration was calculated using a standard curve of sodium nitrite with serial dilution. The reaction volume was 150 µL in total and contained 50 µL of a sample, an equal volume of 1% sulphanilamide in 5% phosphoric acid, and 50 µL of 0.1% napthaylamine diamine dihydrochloric acid in water. Finally, NO was measured at 550 nm using an ELISA reader. 

#### 2.1.4. Synthesis of α-Lipoic Acid Derivatives

New α-LA derivatives and NO-donor/antioxidants bifunctional compounds were synthesized; these compounds will comprise two groups. The first one is based on mono-lipoate, linked to different alkyl spacers, where α-LA is coupled in one side of a short or long flexible spacer and a hydroxyl functional group linked with the second side of the spacer, compounds LA1 and LA4. While the second group, “bifunctional compounds,” is based on α-lipoic acid derivatives as antioxidants and nitric oxide (NO) donors, the α-LA group coupled in one side of a short or long flexible spacer, and the nitric oxide group (NO) conjugated in the other side of the spacer, compounds LA2 and LA3.

**Scheme 1:**
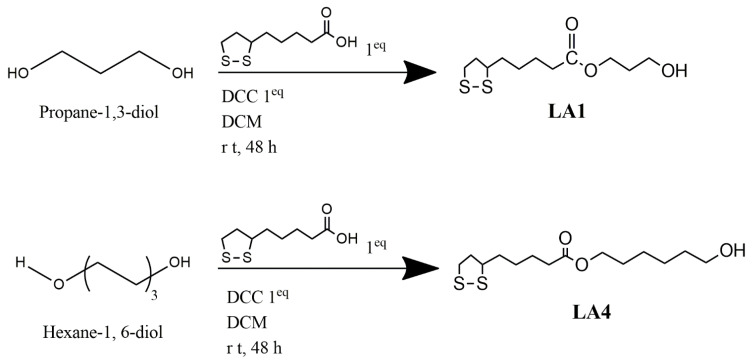
**Synthesis of compounds (LA1 and LA4)**.

**Scheme 2:**
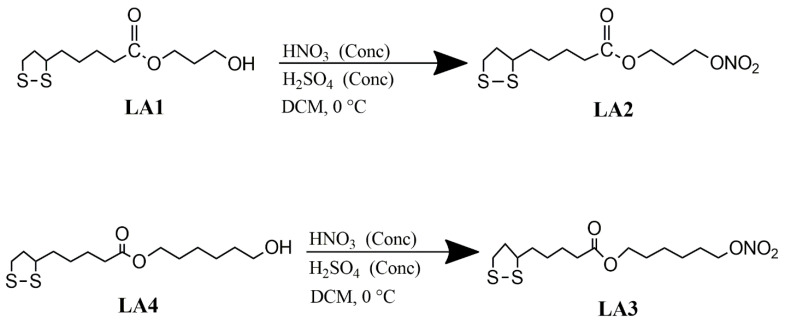
**Synthesis of compounds (LA2 and LA3)**.

Our strategy for synthesizing novel bifunctional compounds, anti-superoxide, and NO donors is based on a unique, simple procedure and good chemistry. The synthesized compounds are summarized in Scheme 1 and Scheme 2, purified by column chromatography (CC) and flash chromatography (FC), and the compounds were characterized and verified by various analytical techniques such as ^1^H NMR and ^13^C NMR spectroscopy, liquid chromatography-mass spectrometry (LC-MS), and infrared spectroscopy (FTIR). The reverse-phase HPLC system determined the purity. A mixture of 90 -100% acetonitrile and 10–0% H_2_O was used as the eluent at a flow rate of 1–3 mL/min, coupled with UV/vis detectors.

Since the nitric oxide donors (compounds LA2 and LA3) are relatively unstable in an aerobic aqueous media, these compounds were characterized by FTIR and ^1^HNMR techniques to show the disappearance of the hydroxyl group, which was replaced with the -ONO_2_ group. The percentage of modification of these compounds was determined by UV spectroscopy based on the Griess Method. The NO release profile compared to its release from standard Na_2_NO_3_ was analyzed by UV spectroscopy based on the Griess Method.

Compounds (LA1 and LA4) shown in Scheme 1 were employed as a starting material to prepare novel compounds, antioxidant/NO donors’ compounds (LA2 and LA3) shown in Scheme 2.

### 2.2. Experimental Section

#### 2.2.1. Characterization of Lipoic Acid

HPLC chromatogram, retention time (r.t) 1.83 min, indicates one compound, meaning that the starting material LA was pure.

^1^H NMR (DMSO-d6) ppm: 7.25 (1H, OH), 3.6 (t, 2H, S-S-CH2), 3.2 (m, 1H, CH2CH-S-S-), 2.45 (t, 2H, -CH2-CO), 2.3 (dt, 2H, -S-S-CH2CH2-),1.9 (m, 2H, -OCO-CH2CH2), 1.7 (dt, 2H, -OCO-CH2CH2CH2CH2-), 1.65 (m, 2H, -OCO-CH2CH2CH2-),

FTIR (cm^−1^, KBr): 2700, 1709.33, 2990,650,550. LC-MS m/z: 206.3 (MH+) (Figure S3).

#### 2.2.2. General Procedure Used for the Synthesis of Compounds LA1 and LA4

##### Synthesis of 1-Hydroxy-3-Lipo Ester (LA-PRO-OH) LA1

DCC (2.058 g, 10.0 mmol) and LA (2.058 g, 10.0 mmol) dissolved in CH_2_Cl_2_ (50 mL) were added to 1, 3-propanediol (1.086 mL, 15 mmol) dissolved in CH_2_Cl_2_ (50 mL) (50 mL). At r t, the mixture was stirred for 48 h. The solution was filtered to remove the precipitated N, N- Di cyclohexyl urea (DCU), and TLC with LA, DCU, and DCC. The remedy was to place it in the refrigerator to extract more DCU. The solution was then filtered once more before being evaporated under reduced pressure. The residue was dissolved in a hot 1:1 EtOH/CH_2_Cl_2_ solution (30 mL) and filtered again.

Following solvent evaporation, the residue was dissolved in a small quantity of CH_2_Cl_2_, and the solution was extracted with 10 mL of 10% NaHCO_3_ and washed with 10 mL of water (twice). The solution was dried with CaCl_2_, and TLC was performed. The solution was evaporated, and the remaining solid was dissolved in a small amount of ethyl acetate, crystallized by ether, putting in the freezer until the crystal was formed; the crystal was filtered with suction filtration and purified by flash column chromatography. HPLC analysis, retention time (r.t) 2.442 min, shows that the compound was pure.

^1^H NMR (DMSO-d6) ppm: 8.3 (1H, OH), 3.9 (t, 2H, -CH_2_OH), 3.4 (t, 2H, S-S-CH_2_), 3.5 (t, 2H, -CH_2_-CO), 3.2 (m, 1H, CH_2_CH-S-S-), 3.1 (t,2H, -CH_2_-CO), 2.5 (dt, 2H, -S-S-CH_2_CH_2_-), 2.2 (m, 2H, -OCO-CH_2_CH_2)_, 1.8 (t, 2H, CH_2_CH_2_OH), 1.7 (dt, 2H, -OCO-CH_2_CH_2_CH_2_CH_2_-), 1.65 (m, 2H, -OCO-CH_2_CH_2_CH_2_-).

^13^C NMR(DMSO-d6) ppm: 173.30, 170.10, 155.01, 64.52, 56.64, 53.14, 50.15, 41.0, 37.30, 32.11, 28.13, 24.68.

FTIR (cm^−1^, KBr): 3296.71, 1697.01, 1229.60, 2929.74, 550, 440.49.

LC-MS m/z: 264.4 (MH^+^) (Figure S4).

##### Synthesis of 1-Hydroxy-6-Lipoester (LA-HEX-OH) LA4

HPLC analysis, retention time (r.t) 2.443 min, analysis indicates one compound.

1H NMR (DMSO-d6) ppm: 8.2 (1H, OH),5.5 (t, 2H, CH_2_OH), 4.0 (m, 2H, CH_2_CH_2_OH), 3.9 (t, 2H, -CH_2_OH), 3.6 (m, 2H, CH_2_CH_2_CH_2_OH), 3.5 (t, 2H, -CH_2_-CO), 3.4 (t, 2H, S-S-CH_2_), 2.2 (m, 2H, -OCO-CH_2_CH_2_), 3.2 (m, 1H, CH_2_CH-S-S-), 3.1 (t,2H, -CH_2_-CO), 2.5 (dt, 2H, -S-S-CH_2_CH_2_-), 1.8 (t, 2H, CH_2_CH_2_OH),1.7 (dt, 2H, -OCO-CH_2_CH_2_CH_2_CH_2_-), 1.65 (m, 2H, -OCO-CH_2_CH_2_CH_2_-).

^13^C NMR(DMSO-d6) ppm: 173.40, 170.09, 154.25, 61.27, 56.72, 53.18, 50.168,

40.45, 34.73, 26.13, 24.88.

FTIR (cm^−1^, KBr):3295.85, 1710.27, 1540.85, 2851, 624, 443.

LC-MS m/z: 306.4 (MH^+^) (Figure S5).

##### Synthesis of Compounds LA2 and LA3

The nitrate esterification of compounds LA2 and LA3 followed the following procedure. The purified and synthesized compounds (LA1 and LA4) were dissolved in CH_2_Cl_2_, adding a 1:1 mixture of HNO_3_ and H_2_SO_4_. The final product was obtained after stirring the mixture for 24 h.

1. 1-Nitrooxy–propane-3-lipoyl ester (LA-PRO-ONO_2_) LA2.

Yield: 92%, modification percent: 95% (Griess Method).

^1^H NMR (DMSO-d6) ppm: 4.01 (t, 2H, -CH 2 ONO 2), 3.7 (m, 2H, -CH 2 CH 2 ONO 2), 3.45 (t, 2H, S-S-CH 2), 3.35 (t, 2H, -CH 2-CO), 3.2 (m, 1H, CH 2 CH-S-S-), 2.45 (m, 2H, -S-S-CH 2 CH 2-), 2.33 (t,2H, -CH 2-CO), 1.55–1.9 (m, 4H, (-CH 2), -OCO-CH 2 CH 2), 1.2–1.4 (m, 2H, -OCO-CH 2 CH 2) (Figure S6).

FTIR (cm^−1^): 3055.22, 2934.72, 1699.35, 1558.10, 1265.42, 740. 32

2. 1-Nitrooxy–Hexane-6-lipoyl ester (LA-HEX-ONO_2_) LA3.

Yield: 89%, modification percent: 97% (Griess Method).

^1^H NMR (DMSO-d6) ppm: 3.9 (t, 2H, -CH 2 ONO 2), 3.6 (m, 2H, CH 2 CH 2 CH 2 ONO 2), 3.45 (t, 2H, S-S-CH 2), 3.4 (t, 2H, -CH 2-CO), 3.2 (m, 1H, CH 2 CH-S-S-), 2.4 (m, 2H, -S-S-CH 2 CH 2-), 2.3 (t,2H, -CH 2 -CO), 1.5–1.8 (m, 8H, (-CH 2) 3, -OCO-CH 2 CH 2), 1.2–1.4 (m, 4H, -OCO-CH 2 CH 2 CH 2 CH 2-, -OCO-CH 2 CH 2 CH 2-) (Figure S7).

FTIR (cm^−1^): 3058.02, 2940.43, 1700.03, 1556.15, 1267.23, 740. 34.

### 2.3. Biological Activity

#### Whole-Cell Patch-Clamp Electrophysiology

Up to 20 μg of high-copy plasmid DNA was prepared using the QIAGEN Plasmid Mini Kit, the tubes were then centrifuged to extract the supernatant containing the plasmid DNA, ethanol was used to wash the DNA pellet, then centrifuged again and the supernatant was carefully removed as to not disturb the pellet, finally, the pellet was air-dried, and the DNA was re-dissolved in a suitable volume of buffer, (our prior study detailed the whole DNA preparation data) [38]. The flip isoform was used in all AMPAR subunits used in this investigation. AMPAR subunits were subcloned in pRK and expressed in the human embryonic kidney cells (HEK293) (The HEK293 cell line was obtained from Sigma, Schnelldorf, Germany). IPA (Integrated Patch Amplifier) recorded HEK293 cells 36–48 h after transfection using the patch-clamp method (Sutter Instruments, Novato, CA, USA). A high-speed piezo solution switcher was installed on a double-barreled glass (theta tube) to provide solutions fast, isolate receptor responses from any network influences, and preserve, as much as possible, the postsynaptic protein machinery. Thus, inhibition was measured by comparing the current recorded when glutamate alone was administered to the current observed when glutamate plus the compound of interest was delivered to the same cell. Before administering the α-LA compounds, experiments were conducted to determine the appropriate concentration to be delivered to the cells without harming them. Dichloromethane (12 µM) effect on GluA1 and GluA2 subunits was tested to ensure that it had no effect on AMPA receptor currents (Figure S8). The effect of α-LA compounds on AMPA subunits plateaued at a concentration of 12 µM because LA inhibits AMPA receptors at low concentrations and continues to inhibit them when LA concentrations are increased by 2 µM each interval, as shown in Figure S1. The concentration increased by 2 µM each time to ensure that no cells were harmed. The cells were washed for 20 s between α-LA derivatives (12 µM), and 10 mM glutamate was supplied alone after each α-LA compound to ensure cell health. Data analysis was carried out using Igor Pro7 (Wave Metrics, inc). For both desensitization (τ_w_ des) and deactivation rates (τ_w_ deact), an exponential fit to the current decline was obtained at 95% of peak to baseline current. The weighted tau (τ_w_) was calculated as τ_w_ = (τf × af) + (τs × as), where af and as are the amplitudes of the fast (τf) and slow (τs) exponential components, respectively. Our study detailed the experimental data set [38,39,40].

### 2.4. Statistical Analysis

Data are presented as mean ± standard error of the mean (SEM). The number of samples (n = 10) refers to the total number of tested HEK293 cells. Statistical differences between the groups and the wild type were examined by One Way Analysis of Variance (ANOVA), with values of *p* < 0.05 being considered to indicate statistical significance: (*) *p* < 0.05, (**) *p* < 0.01, (***) *p* < 0.001, ns, not significant. Concentration-response relationships were fitted as composite curves using GraphPad Prism version 6.01 (GraphPad Software, San Diego, CA, USA) to the Hill equation. All results were representative of at least three independent experiments.

## 3. Results

### 3.1. Modulation of Whole-Cell AMPA Receptor Currents by α-Lipoic Acid

**Figure 2:**
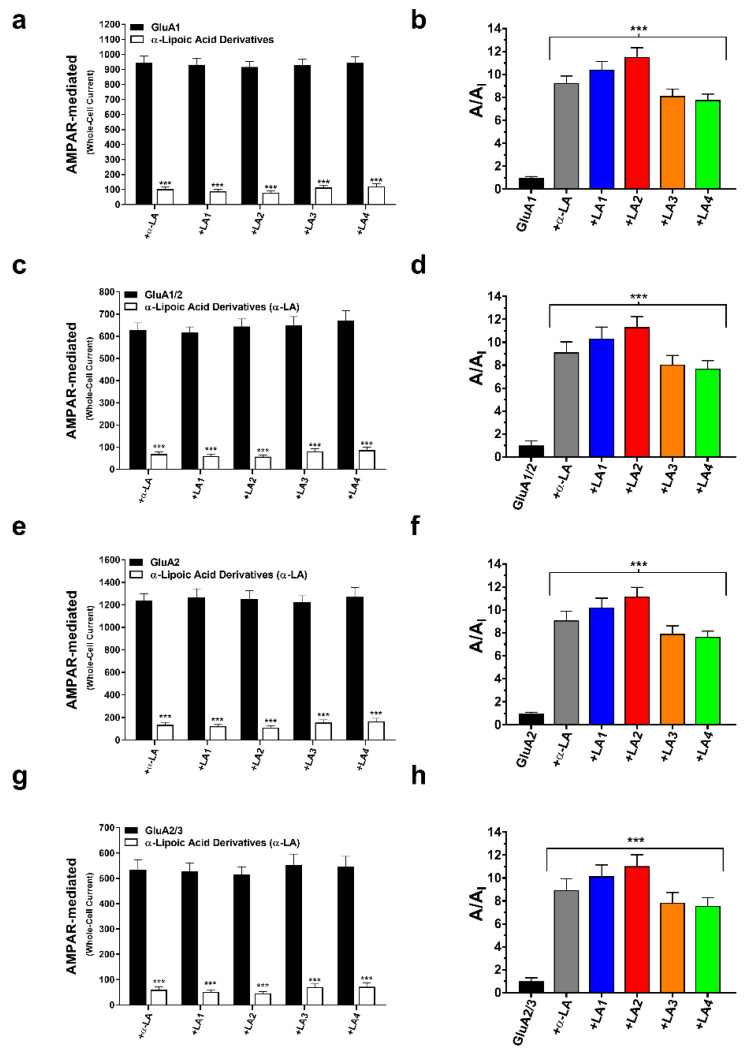
Suppression of AMPA receptor currents by α-LA compounds. Graphs (**a**,**c**,**e**,**g**) present AMPA receptor currents (pA) before and after the administration of α-LA derivatives on GluA1, GluA1/2, GluA2, and GluA2/3 subunits. While (**b**,**d**,**f**,**h**) present the ratio *A/A_I_*, where *A* represents the current caused by 10 mM glutamate alone and *A_I_* represents the current induced by glutamate + α-LA (12 µM). The whole-cell current was measured at −60 mV, pH 7.4, and 22 °C. A one way analysis of variance (ANOVA) was performed for comparison: *** *p* < 0.001; ns, not significant. Ten cells were investigated for each LA compound. All values are shown as mean SEM.

**Figure 3:**
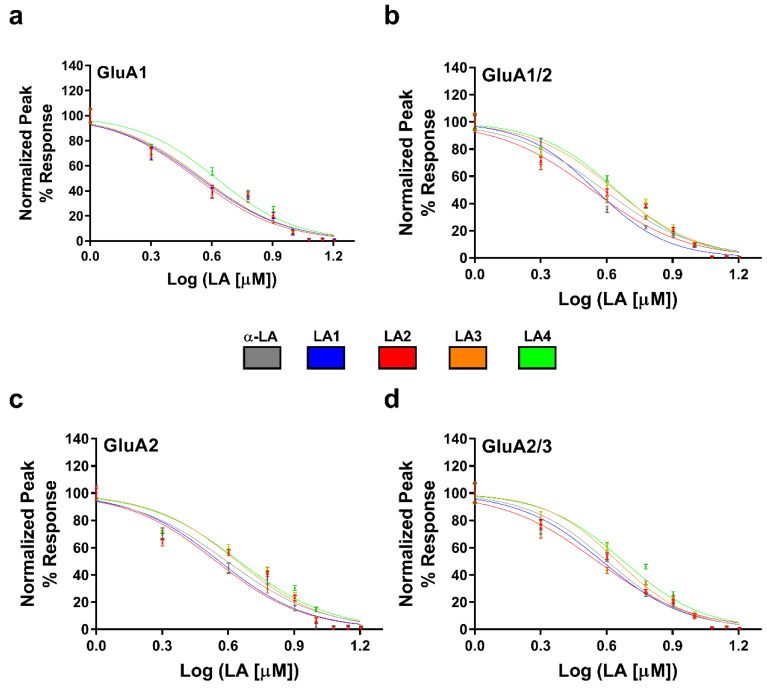
The potency of LA compounds against AMPA receptor subunits. The IC_50_ values for LA derivatives on whole-cell normalized peak amplitude in HEK293 cells are shown in (**a**–**d**). IC_50_ values were calculated using GraphPad Prism version 6.01 (GraphPad Software, San Diego, CA, USA), and concentration-response relationships were fitted as composite curves to the Hill equation. The whole-cell current was measured at −60 mV, pH 7.4, and 22 °C. Each concentration was applied to the cell for 500 ms before rinsing out for 20 ms. Following many washout periods, glutamate (10 mM) was supplied alone, and the instantaneous evoked current was measured. Each data point is the average of three to four different trials.

Our research examined how α-Lipoic Acid (α-LA) affected glutamate-evoked AMPA receptor-mediated whole-cell currents in human embryonic kidney cells (HEK293). Using the whole-cell patch-clamp technique with and without the injection of the α-LA, the homomeric and heteromeric AMPA receptor subunits (i.e., GluA1, GluA2, GluA1/2, and GluA2/3) were investigated (Tables S1–S5). After the injection of α -LA injection, the ratio *A/A_I_*, where *A* represents the current caused by glutamate alone, and *A_I_* represent the current induced by glutamate + α-LA, increased by 9-fold (Figure 2a,c,e,g). α-LA inhibited AMPA receptor-mediated responses with an approximate IC_50_ value of 3.5–4 μM, indicating that it was potent against AMPA receptor subunits (Table S6, Figure 3a–d).

### 3.2. α-LA Derivatives (LA1, LA2, LA3, and LA4) Reduced AMPA Receptor Activation While Enhancing the Deactivation of AMPA Receptor Subunits

**Figure 4:**
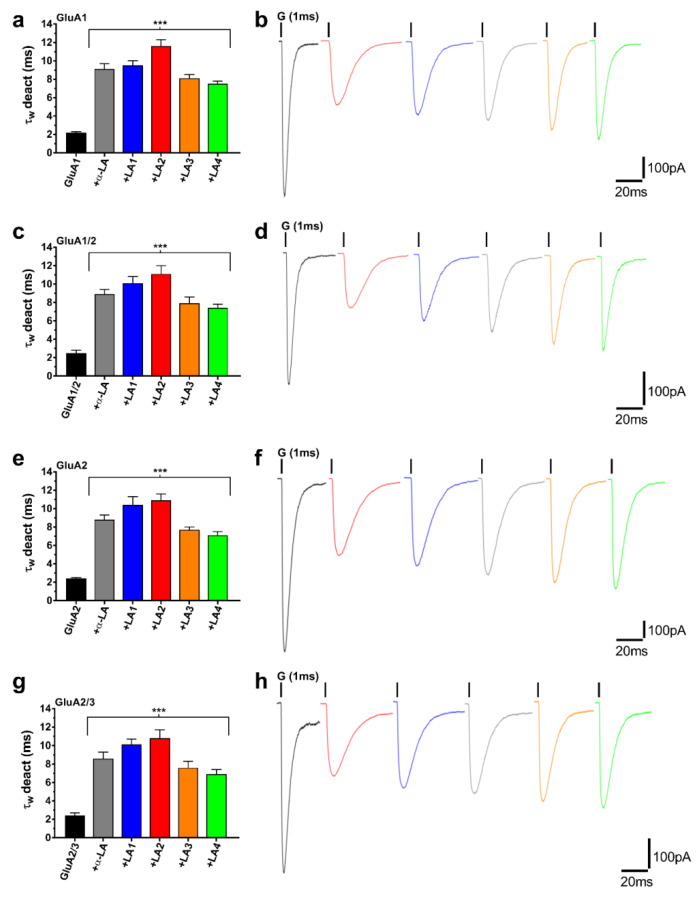
The effects of α-LA and its derivatives on AMPA receptor deactivation rate. As shown in the graphs, each subunit was influenced by the α-LA derivatives, with (**a**) representing the effect of α-LA derivatives on GluA1 deactivation rate, (**c**) representing the impacts of α-LA derivatives on GluA1/2, (**e**) representing the effects of α-LA derivatives on GluA2, and (**g**) demonstrating the effect of α-LA derivatives on the deactivation rate of GluA2/3. α-LA derivatives significantly affected AMPA receptor subunits, as seen by the traces in (**b**,**d**,**f**,**h**). The whole-cell current was measured at −60 mV, pH 7.4, and 22 °C. A one way analysis of variance (ANOVA) was performed for comparison: *** *p* < 0.001; ns, not significant. Ten cells were investigated for each LA compound. As stated in the supplementary material tables, the cells were washed for 20 s between α-LA compounds, and a 10 mM glutamate was supplied alone after each α-LA compound (12µM) to ensure cell health. All values are shown as mean SEM.

Four novel LA derivatives were synthesized and tested on AMPA receptor subunits using the α-LA molecule as a template. When the LA2 chemical was delivered to the cell, it resulted in a nearly 11-fold decrease in the peak current of the AMPARs. Likewise, the action of LA1 had a similar influence on the peak current of AMPAR subunits by about 10-fold. LA3 and LA4 lowered AMPA receptor currents by roughly 8-fold (Figure 2b,d,f,h). Hence, the peak currents of GluA1, GluA2, GluA1/2, and GluA2/3 were lowered considerably by α-LA and its derivatives. LA1 inhibited AMPA receptor subunits with approximate IC_50_ values of 3.5–3.8 μM, while LA2 reduced AMPA receptor currents with an approximate IC_50_ value of 3.5-μM, and LA3 and LA4 affected AMPA receptor subunits with approximate IC_50_ values of 4–4.7 μM (Figure 3, Table S6). However, α-LA and its derivatives increased the rate of AMPA receptor deactivation (τ_w_ deact), implying that the deactivation time course was slowed because the rate is the inverse of the time constant (Figure 4a,c,e,g). The findings revealed that -LA and LA1 increased the rate by about 4-fold. At the same time, LA2 increased (τ_w_ deact) approximately 5-fold. LA3 and LA4 influenced the deactivation rate roughly 3-fold (Figure 4b,d,f,h).

### 3.3. Concentration-Dependent Inhibition of the Derivatives on AMPA-Type Receptors

The levels of inhibition of α-LA and its derivatives attracted our attention to assessing the potency of the compounds in a concentration-dependent manner. Recording inhibition of different antagonist doses demonstrated the maximal efficacy and the plateau concentration (14 μM), as seen in Figure S1. The concentration−inhibition curves were produced by administering glutamate alone at a concentration of 10 mM, followed by glutamate + α-LA derivatives at varying concentrations (in μM) for every recording point. Afterward, the cells were normalized back by administering glutamate alone. Since the glutamate concentration in the synaptic cleft constantly saturates the receptors, driving the response to a near maximum level, variability occurs because the maximum level changes. The maximum capability of the postsynaptic receptor ensemble can change due to various factors, such as desensitization by the previously released transmitter. So the use of a saturating glutamate concentration leads to open most of the channel population and will be in the open conformation.

On the other hand, increasing the glutamate concentration (2 µM) each time induces the closed channels to open and reach the maximum activity. As a result, while testing the same LA compound, it was essential to use different glutamate concentrations and to increase it gradually to reach the maximally activated state, as the channels remain in a particular mode for a longer time than seen in lower concentrations of glutamate. This study noticed that the inhibition of AMPAR subunits by the LA compounds (12 µM) was independent of glutamate concentrations and could not be surmounted by increasing glutamate concentrations (Figure S2).

### 3.4. α-LA and Its Derivatives Reduced AMPA Receptor Desensitization Rates (τ_w_ des)

**Figure 5:**
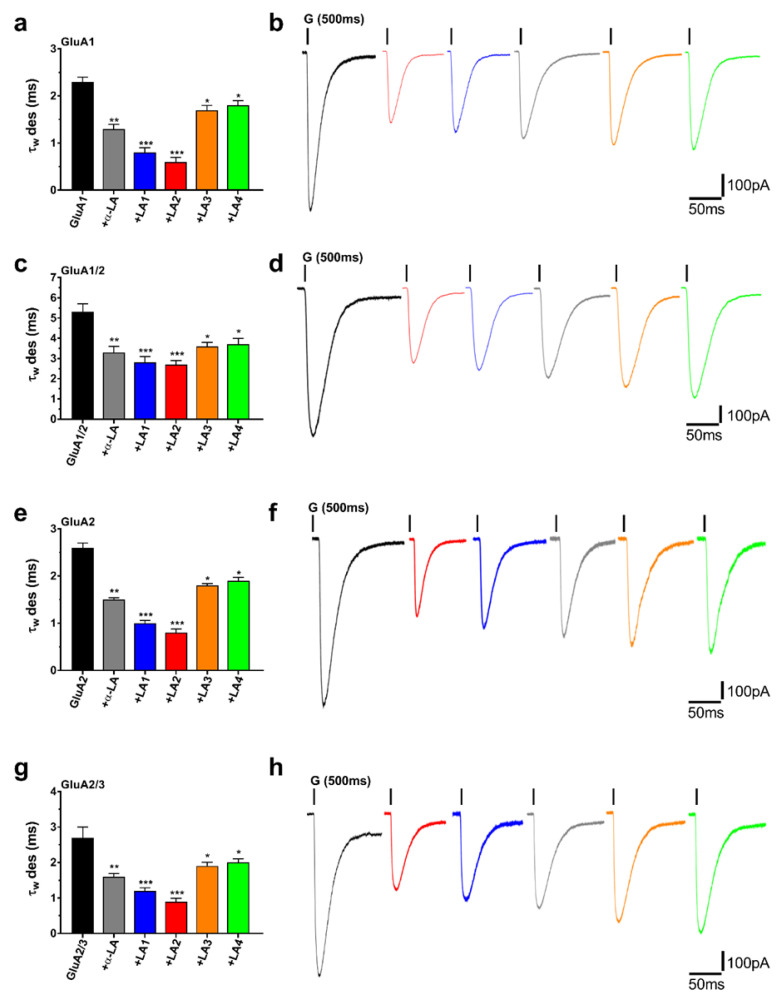
The effects of α-LA derivatives on AMPA receptor desensitization rate. As shown in the graphs, each subunit was influenced by the α-LA derivatives, with (**a**) representing the effect of α-LA derivatives on GluA1 desensitization rate, (**c**) representing the impacts of α-LA derivatives on GluA1/2, (**e**) representing the effects of α-LA derivatives on GluA2, and (**g**) demonstrating the effect of α-LA derivatives on the desensitization rate of GluA2/3. The traces in (**b**,**d**,**f**,**h**) show that α-LA derivatives substantially impacted AMPA receptor subunits. The whole-cell current was measured at −60 mV, pH 7.4, and 22 °C. A one way analysis of variance (ANOVA) was performed for comparison: * *p* < 0.05; ** *p* < 0.01; *** *p* < 0.001; ns, not significant. Ten cells were investigated for each LA derivative. The cells were rinsed for 20 s between α-LA compounds, and a 10 mM glutamate was administered alone after each α-LA compound (12 µM) to guarantee cell health, as shown in the supplementary material tables. All values are shown as mean SEM.

AMPA receptor subunits were expressed in HEK293 cells to gain more control over the discovered AMPA receptor desensitization rates (τ_w_ des), and recordings were made using the patch-clamp method in conjunction with a fast perfusion system. As demonstrated in Figure 5a,c,e,g, AMPA receptor subunits quickly desensitized when glutamate alone was applied. At the same time, a cell treated with glutamate plus α-LA desensitized faster due to a 2-fold reduction in the rate of desensitization (τ_w_ des). In comparison, LA1 and LA2 lowered the (τ_w_ des) by roughly 3–4-fold. In comparison, LA3 and LA4 demonstrated a fold reduction in AMPA receptor desensitization rate (Figure 5b,d,f,h).

## 4. Discussion

This research’s main objective is to enhance lipoic acid’s effectiveness with some modifications to its general structure. Therefore, several new lipoic acid derivatives have been prepared to compare their biological activity with lipoic acid on AMPA receptors. The evaluated compounds, including lipoic acid, were classified into three types: The first one is α-lipoic acid, which contains a disulfide group on one side and a carboxylic group on the other, the second type is LA1, and LA4 contains a disulfide group on one side and a hydroxide group on the other. The third type is LA2, and LA3 contains a disulfide group on one side and a nitrate ester (-ONO_2_) group on the second. In its components and action, this new and innovative molecule operates as both antioxidants and nitric oxide donors in one pot (bifunctional NO-donor/antioxidant).

According to the findings, LA1 and LA2 have more significant potential on AMPA receptors than α-lipoic acid. When the activities of the compounds LA1 and LA2 were compared, it was discovered that compound LA2 was more potent than compound LA1. These findings support the concept that we used to create the dual-active chemical. When the activity of LA3 and LA4 is compared, the findings support our prediction that the bifunctional compound LA3 (NO-donor/antioxidant) significantly impacts AMPA receptors more than compound LA4. Interestingly, compound LA2 is very similar to compound LA3, with a difference in the number of hydrocarbons between the two functional groups. At the same time, the activity of LA2 was greater than that of LA4, which means it is necessary to conduct other experiments to confirm the influence of the length of the hydrocarbon chain on the effectiveness.

In terms of the effects of the α-LA on the central nervous system (CNS), preclinical studies have demonstrated that this compound has anti-inflammatory properties and can prevent neuronal damage caused by a pathophysiological alteration observed in neurodegenerative diseases such as Alzheimer’s disease. α-LA also can influence nitric oxide levels in the brain and neural tissue, which might have implications for neurodegeneration, learning, cognition, and the process of aging. α-LA is already employed as a therapeutic agent in treating many neurological conditions, including diabetic neuropathy and polyneuritis, in which patients’ endogenous α-LA levels have been depleted or absent. Since enzymatic antioxidants are involved in the majority of the brain’s defense mechanisms, it is possible that supplementing the brain with antioxidants such as α-LA could be a therapeutic approach to reduce oxidative stress and aid in the treatment or prevention of age-associated memory impairment and neurodegenerative disorders such as Alzheimer’s disease. In addition, the abundance, trafficking, localization, and functions of AMPA receptors have all been dysregulated across various Alzheimer’s disease models. As a result, our findings were of particular interest since α-LA derivatives, when used to modulate AMPA receptors, may aid research into regulating Alzheimer’s disease and various other clinical conditions, including autism spectrum disorders, Parkinson’s disease, and epilepsy.

α-LA derivatives may inhibit AMPA receptors via interacting with allosteric AMPA binding sites since increasing the glutamate concentration had no effect on the inhibitory action of the α-LA derivatives (Figure S2), showing that the α-LA binds AMPA receptors allosterically. Multiple negative allosteric modulators of AMPA receptors have been developed in recent years, which is significant. GYKI 52,466 was one of the first modulators of AMPA receptors [39], and it was used as a model for developing new, more powerful, and less toxic AMPAR modulators. Several derivatives have been synthesized by adding alternative functions to the 2,3-benzodiazepine system, resulting in anticonvulsant medications that are more active, less toxic, and last longer. Numerous studies have found that several of these compounds contribute to the same binding pocket. Residues in the pre-M1 (Ser 516, Phe 517, Asp 519, Pro 520), M3 (Ser 615, Tyr 616, Leu 620, Phe 623), and M4 (Asn 791) helices of the receptor, as well as one residue in the S2-M4 (Ser 788) linker, are among those targeted. The S2-M4 linker, in particular, demonstrates remarkable adaptability since two neighboring pockets may share it, allowing them to interact with two ligands simultaneously [41,42].

Several studies have been conducted on chemicals and their effects on AMPA receptors, whereas α-LA derivatives have been tested on homomeric and heteromeric AMPA receptor subunits. Lipoic acid and its derivatives inhibited both homomeric and heteromeric AMPA receptor subunits, which was critical for our study since both subunits are involved in AMPA receptor activity. While most AMPARs appear to be heteromeric GluA2-containing assemblies with low calcium permeability, GluA2-deficient, calcium-permeable AMPARs constitute a sizable and crucial minority that play critical roles in synaptic signaling, learning, and pathology. Furthermore, the biophysical gating properties of the AMPA receptor (i.e., deactivation and desensitization) were modified by the α-LA compound and its derivatives in this study. Since it can potentially change the pattern of synaptic transmission, control the activity of postsynaptic receptors, and shield neurons from the neurotoxic effects of glutamate, the role of desensitization is gaining considerable attention. The gating features of AMPA receptor channels and the individual excitatory postsynaptic currents are still not completely understood. As a result, researching the influence of α-LA on desensitization and deactivation rates may be beneficial in understanding how AMPA receptors behave.

α-LA and its derivatives have strong neuroprotective effects on AMPA receptor subunits. However, the evidence is limited primarily due to the small number of studies on α-LA on AMPA receptors since our work is the first to investigate the neuroprotective impact of α-LA compounds on AMPA receptors. Furthermore, a lack of knowledge of the AMPA receptor’s structure makes it impossible to anticipate how α-LA will bind directly to AMPA and what precise conformational changes will occur.

## 5. Conclusions

Recent research demonstrated that the modulation of ionotropic neurotransmitter receptors is crucial for controlling synaptic efficiency and underpinning numerous types of synaptic plasticity. The experimental findings confirm the notion that α-Lipoic acid has multiple effects on diverse parts of the brain and has been studied in several studies of aging and degenerative disorders as the tested compounds significantly inhibited AMPA receptor currents after evaluating the effects of the five LA derivatives on AMPA receptor subunits (i.e., GluA1, GluA1/2, GluA2, and GluA2/3) and obtaining electrophysiology recordings. The five LA derivatives tested had a significant impact on the biophysical gating properties of AMPA receptors (desensitization and deactivation), giving the LA derivatives a high value and making them extremely promising for use in AMPA receptor modulation and, as a result, the treatment of a wide range of neurodegenerative diseases.

## Data Availability

All data generated or analyzed during this study are included in this published article [and its supplementary material files].

## Supplementary Materials

The following supporting information can be downloaded at: https://www.mdpi.com/article/10.3390/cells11223608/s1, Figure S1: Concentration-dependent inhibition of α-LA derivatives on AMPA-type receptors. Graphs a-d show the cell was treated with glutamate (Glu) alone, Glu + LA compounds (μM) using the 500 ms protocol, and Glu alone to guarantee cell health. The cell was washed for 10 ms after each concentration increment to allow it to recover completely. Following multiple washout periods, the remaining recording points were collected using the same approach, with a 2 μM increase between each concentration point until 20 μM. Data points (n = 10) were normalized to the response of different LA compound concentrations; Figure S2: Dose-dependent inhibition Curve for AMPAR-type Subunits. a-d. Demonstrate the dose-dependent glutamate effect on AMPAR subunits by treating the cells independently with α-LA and its derived compounds at varied glutamate doses (2–12 µM). The ratio A/AI for all compounds reaches a plateau at 2 µM, where A represents the normal current of the AMPA receptor and AI represents the inhibitory current of the AMPA receptor and remains constant as Glu concentration increases. Consequently, altering the glutamate concentrations did not influence the LA derivatives effect. The glutamate concentrations steadily climbed from 2 µM to 12 µM as the LA derivatives were applied to the subunit. The whole-cell current was measured at −60 mV, pH 7.4, and 22 °C; Figure S3: Characterization of Lipoic acid; Figure S4: Characterization of Lipoic acid of 1-hydroxy-3- lipo ester (LA-PRO-OH) LA1; Figure S5: Characterization of 1-hydroxy-6-lipoester (LA-HEX-OH) LA4; Figure S6: Characterization of Nitrooxy–propane-3-lipoyl ester (LA-PRO-ONO2) LA2; Figure S7: Characterization of 1-Nitrooxy–Hexane-6-lipoyl ester (LA-HEX-ONO2) LA3; Figure S8: Effect of dichloromethane on GluA1 and GluA2 subunits. Graphs a–d demonstrates the effects of dichloromethane on GluA1 and GluA2 whole-cell current, as well as the rates of deactivation and desensitization. Cells were treated with 12 µM dichloromethane at a concentration comparable to that of the LA derivatives; Table S1: Whole-Cell Recordings for Compound α-LA, ** *p* < 0.01; *** *p* < 0.001; Table S2: Whole-Cell Recordings for Compound LA1, *** *p* < 0.001; Table S3: Whole-Cell Recordings for Compound LA2, *** *p* < 0.001; Table S4: Whole-Cell Recordings for Compound LA3, * *p* < 0.05; *** *p* < 0.001; Table S5: Whole-Cell Recordings for Compound LA4, * *p* < 0.05; *** *p* < 0.001; Table S6: IC_50_ values.

## Abbreviations

AMPA	α-amino-3-hydroxy-5-methyl-4-isoxazole propionic acid
α-LA	alpha-Lipoic acid
HEK293	Human embryonic kidney 293 cells
AMPARs	α-amino-3-hydroxy-5-methyl-4-isoxazole propionic acid receptors
ATD	amino-terminal domain
LBD	ligand-binding domain
TMD	transmembrane domain
CTD	carboxyl-terminal domain
PAMs	positive allosteric modulators
NAMs	negative allosteric modulators
DCC	Dicyclohexylcarbodiimide
DCM	dichloromethane
NO	Nitric oxide
CC	column chromatography
FC	flash chromatography
LC-MS	liquid chromatography-mass spectrometry
DCU	Di cyclohexyl urea
CNS	central nervous system.
