# Supplementary material for: α-Lipoic Acid Derivatives as Allosteric Modulators for Targeting AMPA-Type Glutamate Receptors’ Gating Modules
Source: Cells. 2022 Nov 15;11(22):3608. doi: 10.3390/cells11223608 (PMC9688225; doi:10.3390/cells11223608)
Supplement: Supplementary file 1 [file cells-11-03608-s001.zip › cells-2035724-supplementary.pdf]

<u>Supplemental Table S1. Whole-Cell Recordings for Compound α-LA</u>

** $p < 0.01$; *** $p < 0.001$

Receptor Name/Compounds abbreviation	GluA1 (Glutamate Alone)	-Lipoic Acid	Applying Glutamate Alone After a-Lipoic Acid	n	A/A_I
**Amplitude (pA)**	943±45	102±14.0***	941±44	10	9.26±0.6
**t deact (ms)**	2.2±0.1	9.1±0.6***	N/R	10	N/R
**t des (ms)**	2.3±0.1	1.3±0.1**	N/R	10	N/R
**Receptor Name/Compounds abbreviation**	**GluA1/2 (Glutamate Alone)**	**-Lipoic Acid**	**Applying Glutamate Alone After a-Lipoic Acid**	**n**	**A/A_I**
**Amplitude (pA)**	629±31	69±10.0***	627±30	10	9.13±0.9
**t deact (ms)**	2.5±0.3	8.9±0.5***	N/R	10	N/R
**t des (ms)**	5.3±0.4	3.3±0.3**	N/R	10	N/R
**Receptor Name/Compounds abbreviation**	**GluA2 (Glutamate Alone)**	**-Lipoic Acid**	**Applying Glutamate Alone After a-Lipoic Acid**	**n**	**A/A_I**
**Amplitude (pA)**	1237±62	136±22***	1235±60	10	9.09±0.8
**t deact (ms)**	2.4±0.1	8.8±0.5***	N/R	10	N/R
**t des (ms)**	2.6±0.1	1.5±0.04**	N/R	10	N/R

Receptor Name/Compounds abbreviation	GluA2/3 (Glutamate Alone)	-Lipoic Acid	Applying Glutamate Alone After a-Lipoic Acid	n	A/A$_I$
**Amplitude (pA)**	535±37	60±11.0***	532±35	10	8.93±1.0
**t deact (ms)**	2.4±0.3	8.6±0.7***	N/R	10	N/R
**t des (ms)**	2.7±0.3	1.6±0.09**	N/R	10	N/R

Supplemental Table S2. Whole-Cell Recordings for Compound LA1

*** $p < 0.001$

Receptor Name/Compounds abbreviation	GluA1 (Glutamate Alone)	LA1	Applying Glutamate Alone After LA1	n	A/A$_I$
**Amplitude (pA)**	931±41	89±12.0***	929±39	10	10.43±0.7
**t deact (ms)**	N/R	9.5±0.5***	N/R	10	N/R
**t des (ms)**	N/R	0.8±0.1***	N/R	10	N/R

Receptor Name/Compounds abbreviation	GluA1/2 (Glutamate Alone)	LA1	Applying Glutamate Alone After LA1	n	A/A$_I$
**Amplitude (pA)**	616±26	60±9.0***	618±27	10	10.32±1.0
**t deact (ms)**	N/R	10.1±0.7***	N/R	10	N/R
**t des (ms)**	N/R	2.8±0.3***	N/R	10	N/R

Receptor Name/Compounds abbreviation	GluA2 (Glutamate Alone)	LA1	Applying Glutamate Alone After LA1	n	A/AI
**Amplitude (pA)**	1264±78	124±19.0***	1262±76	10	10.22±0.8
**t deact (ms)**	N/R	10.4±0.9***	N/R	10	N/R
**t des (ms)**	N/R	1.0±0.06***	N/R	10	N/R
**Receptor Name/Compounds abbreviation**	**GluA2/3 (Glutamate Alone)**	**LA1**	**Applying Glutamate Alone After LA1**	**n**	**A/AI**
**Amplitude (pA)**	527±33	52±8.0***	529±34	10	10.14±1.0
**t deact (ms)**	N/R	10.1±0.6***	N/R	10	N/R
**t des (ms)**	N/R	1.2±0.09***	N/R	10	N/R

Supplemental Table S3. Whole-Cell Recordings for Compound LA2

*** $p < 0.001$

Receptor Name/Compounds abbreviation	GluA1 (Glutamate Alone)	LA2	Applying Glutamate Alone After LA2	n	A/AI
**Amplitude (pA)**	915±37	79±11.0***	917±38	10	11.53±0.8
**t deact (ms)**	N/R	11.6±0.7***	N/R	10	N/R
**t des (ms)**	N/R	0.6±0.1***	N/R	10	N/R

Receptor Name/Compounds abbreviation	GluA1/2 (Glutamate Alone)	LA2	Applying Glutamate Alone After LA2	n	A/A_I
**Amplitude (pA)**	644±34	57±8.0***	642±33	10	11.34±0.9
**t deact (ms)**	N/R	11.1±0.9***	N/R	10	N/R
**t des (ms)**	N/R	2.7±0.2***	N/R	10	N/R

Receptor Name/Compounds abbreviation	GluA2 (Glutamate Alone)	LA2	Applying Glutamate Alone After LA2	n	A/A_I
**Amplitude (pA)**	1253±71	112±17.0***	1250±69	10	11.16±0.7
**t deact (ms)**	N/R	10.9±0.7***	N/R	10	N/R
**t des (ms)**	N/R	0.8±0.08***	N/R	10	N/R

Receptor Name/Compounds abbreviation	GluA2/3 (Glutamate Alone)	LA2	Applying Glutamate Alone After LA2	n	A/A_I
**Amplitude (pA)**	514±31	46±7.0***	515±30	10	11.03±1.0
**t deact (ms)**	N/R	10.8±0.9***	N/R	10	N/R
**t des (ms)**	N/R	0.9±0.09***	N/R	10	N/R

Supplemental Table S4. Whole-Cell Recordings for Compound LA3

* $p < 0.05$; *** $p < 0.001$

Receptor Name/Compounds abbreviation	GluA1 (Glutamate Alone)	LA3	Applying Glutamate Alone After LA3	n	A/A_I
**Amplitude (pA)**	929±38	114±12.0***	930±39	10	8.13±0.6
**t deact (ms)**	N/R	8.1±0.4***	N/R	10	N/R
**t des (ms)**	N/R	1.7±0.1*	N/R	10	N/R
**Receptor Name/Compounds abbreviation**	**GluA1/2 (Glutamate Alone)**	**LA3**	**Applying Glutamate Alone After LA3**	**n**	**A/A_I**
**Amplitude (pA)**	651±38	81±12.0***	652±40	10	8.05±0.8
**t deact (ms)**	N/R	7.9±0.67***	N/R	10	N/R
**t des (ms)**	N/R	3.6±0.2*	N/R	10	N/R
**Receptor Name/Compounds abbreviation**	**GluA2 (Glutamate Alone)**	**LA3**	**Applying Glutamate Alone After LA3**	**n**	**A/A_I**
**Amplitude (pA)**	1223±57	155±25***	1225±58	10	7.91±0.7
**t deact (ms)**	N/R	7.7±0.3***	N/R	10	N/R
**t des (ms)**	N/R	1.8±0.04*	N/R	10	N/R

Receptor Name/Compounds abbreviation	GluA2/3 (Glutamate Alone)	LA3	Applying Glutamate Alone After LA3	n	A/AI
Amplitude (pA)	553±43	71±14.0***	554±44	10	7.84±0.9
t deact (ms)	N/R	7.6±0.7***	N/R	10	N/R
t des (ms)	N/R	1.9±0.1*	N/R	10	N/R

Supplemental Table S5. Whole-Cell Recordings for Compound LA4

* $p < 0.05$; *** $p < 0.001$

Receptor Name/Compounds abbreviation	GluA1 (Glutamate Alone)	LA4	Applying Glutamate Alone After LA4	n	A/AI
Amplitude (pA)	946±39	121±18***	947±38	10	7.79±0.5
t deact (ms)	N/R	7.5±0.3***	N/R	10	N/R
t des (ms)	N/R	1.8±0.1*	N/R	10	N/R
**Receptor Name/Compounds abbreviation**	**GluA1/2 (Glutamate Alone)**	**LA4**	**Applying Glutamate Alone After LA4**	**n**	**A/AI**
Amplitude (pA)	671±45	87±13.0***	669±44	10	7.70±0.7
t deact (ms)	N/R	7.4±0.4***	N/R	10	N/R
t des (ms)	N/R	3.7±0.3*	N/R	10	N/R

Receptor Name/Compounds abbreviation	GluA2 (Glutamate Alone)	LA4	Applying Glutamate Alone After LA4	n	A/A_I
**Amplitude (pA)**	1270±82	166±28***	1268±78	10	7.65±0.5
**t deact (ms)**	N/R	7.1±0.4***	N/R	10	N/R
**t des (ms)**	N/R	1.9±0.07*	N/R	10	N/R
**Receptor Name/Compounds abbreviation**	**GluA2/3 (Glutamate Alone)**	**LA4**	**Applying Glutamate Alone After LA4**	**n**	**A/A_I**
**Amplitude (pA)**	547±40	72±15.0***	549±43	10	7.58±0.7
**t deact (ms)**	N/R	6.9±0.5***	N/R	10	N/R
**t des (ms)**	N/R	2.0±0.1*	N/R	10	N/R

Supplemental Table S6. IC_{50} values

**Receptor/Subunit**	**_Lipoic Derivatives**	-LA	LA1	LA2	LA3	LA4
**GluA1**	**IC_{50}**	3.54	3.47	3.35	3.59	4.18
	**Log IC_{50}**	0.55	0.54	0.53	0.56	0.62
	**R square**	0.96	0.96	0.97	0.96	0.97

Receptor/Subunit	**_Lipoic Derivatives**	-LA	LA1	LA2	LA3	LA4
**GluA1/2**	**IC_{50}**	3.86	3.54	3.41	4.33	4.41
	**Log IC_{50}**	0.59	0.55	0.53	0.64	0.64
	**R square**	0.97	0.99	0.98	0.97	0.98

Receptor/Subunit	**_Lipoic Derivatives**	-LA	LA1	LA2	LA3	LA4
**GluA2**	**IC_{50}**	3.93	3.63	3.51	4.37	4.47
	**Log IC_{50}**	0.59	0.56	0.55	0.64	0.65
	**R square**	0.93	0.96	0.97	0.94	0.95

Receptor/Subunit	**_Lipoic Derivatives**	-LA	LA1	LA2	LA3	LA4
**GluA2/3**	**IC_{50}**	4.01	3.80	3.61	4.46	4.76
	**Log IC_{50}**	0.60	0.58	0.56	0.65	0.68
	**R square**	0.99	0.98	0.97	0.99	0.96

Figure S1. Concentration-dependent inhibition of α-LA derivatives on AMPA-type receptors. Graphs a-d show the cell was treated with glutamate (Glu) alone, Glu + LA compounds (μM) using the 500 ms protocol, and Glu alone to guarantee cell health. The cell was washed for 10 ms after each concentration increment to allow it to recover completely. Following multiple washout periods, the remaining recording points were collected using the same approach, with a 2 μM increase between each concentration point until 20 μM. Data points (n = 10) were normalized to the response of different LA compound concentrations.

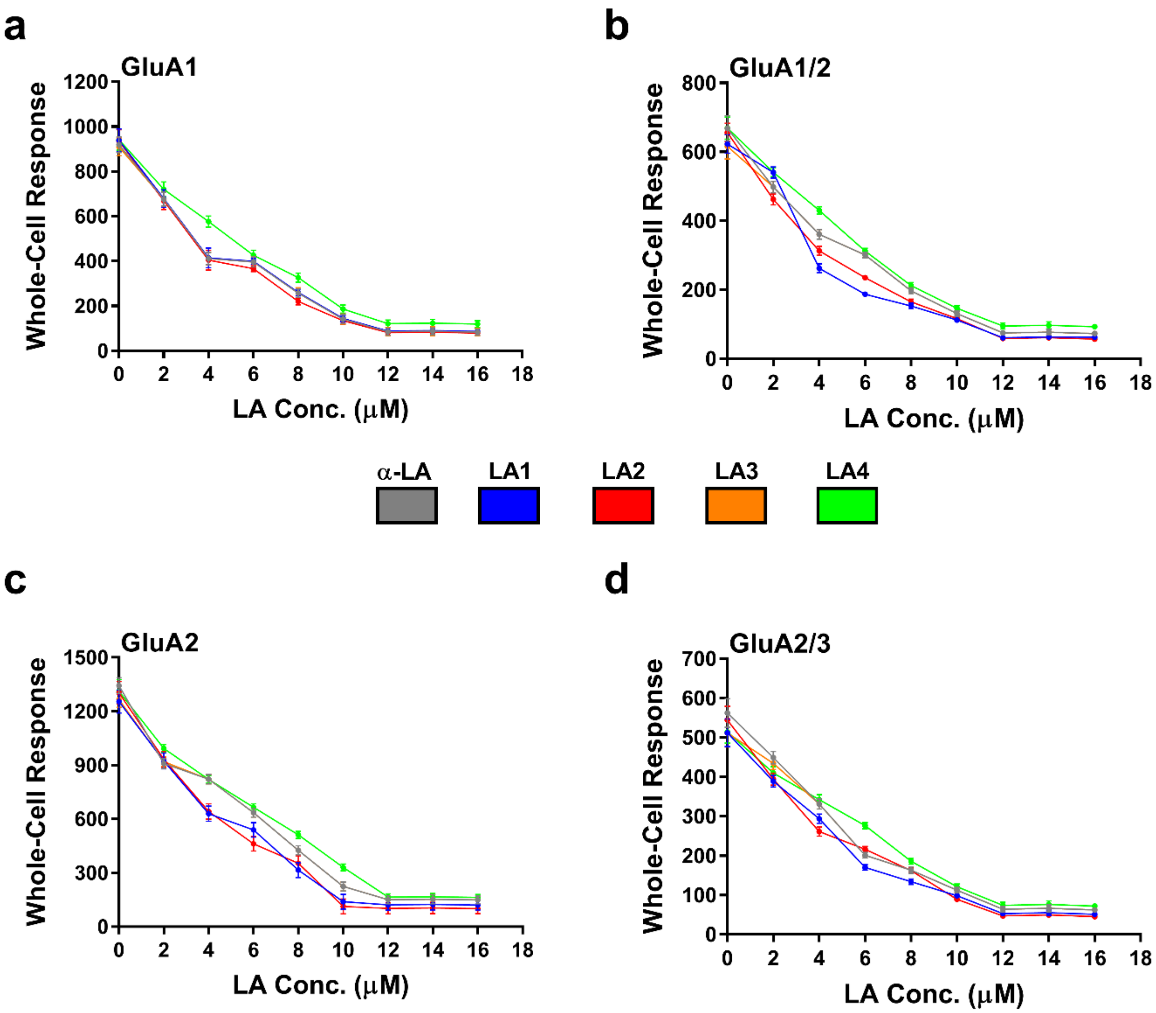

<u>Figure S2. Dose-dependent inhibition Curve for AMPAR-type Subunits</u>. a-d. Demonstrate the dose-dependent glutamate effect on AMPAR subunits by treating the cells independently with α-LA and its derived compounds at varied glutamate doses (2-12 μM). The ratio A/A_I for all compounds reaches a plateau at 2 μM, where A represents the normal current of the AMPA receptor and A_I represents the inhibitory current of the AMPA receptor and remains constant as Glu concentration increases. Consequently, altering the glutamate concentrations did not influence the LA derivatives effect. The glutamate concentrations steadily climbed from 2 μM to 12 μM as the LA derivatives were applied to the subunit. The whole-cell current was measured at −60 mV, pH 7.4, and 22 °C.

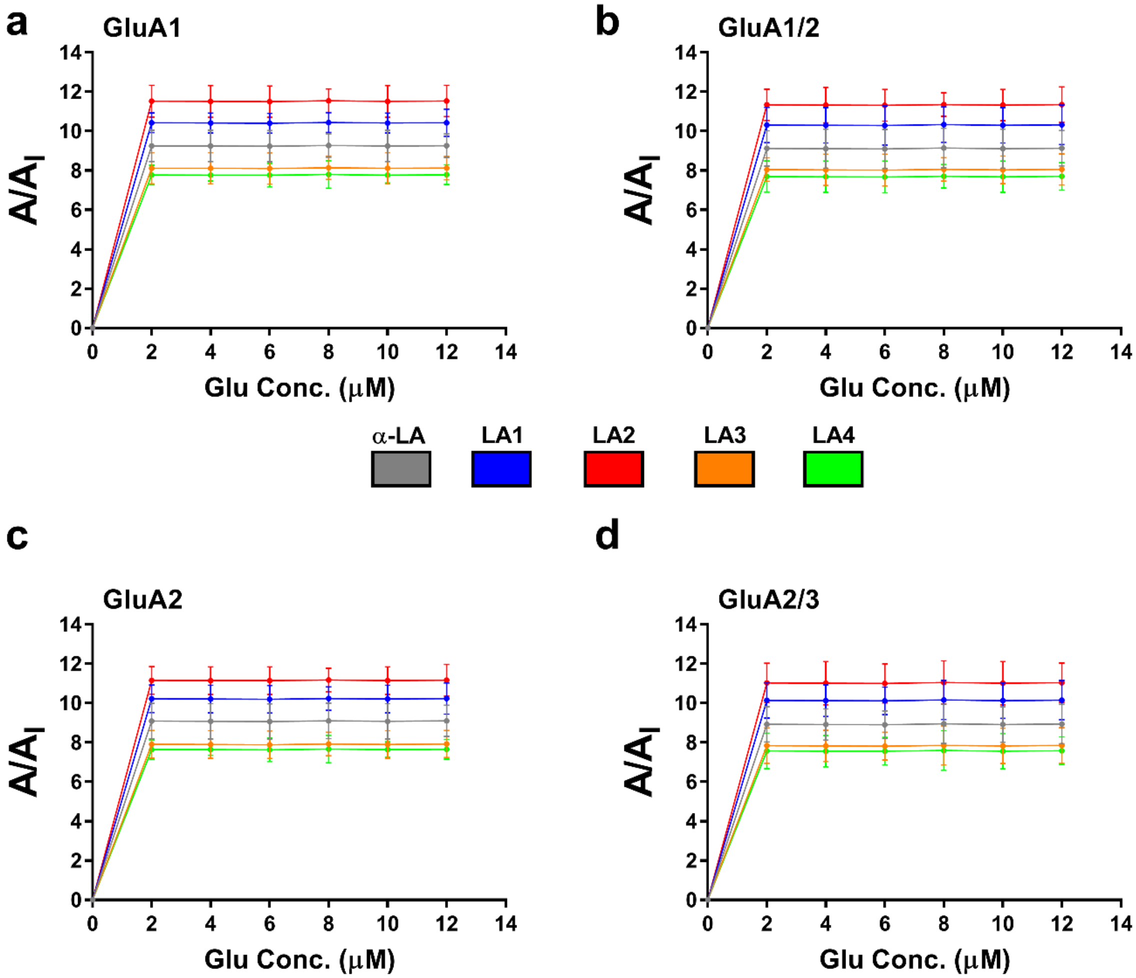

Some selected spectroscopic spectrum analyses were used for the characterization and identification of the synthesized compounds

<u>Figure S3. Characterization of Lipoic acid</u>

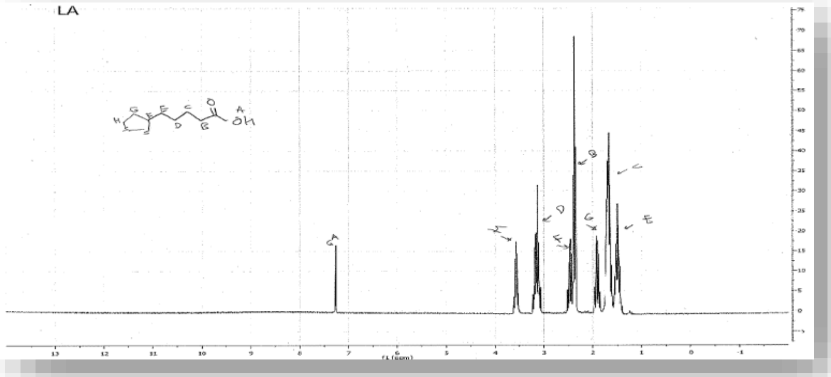

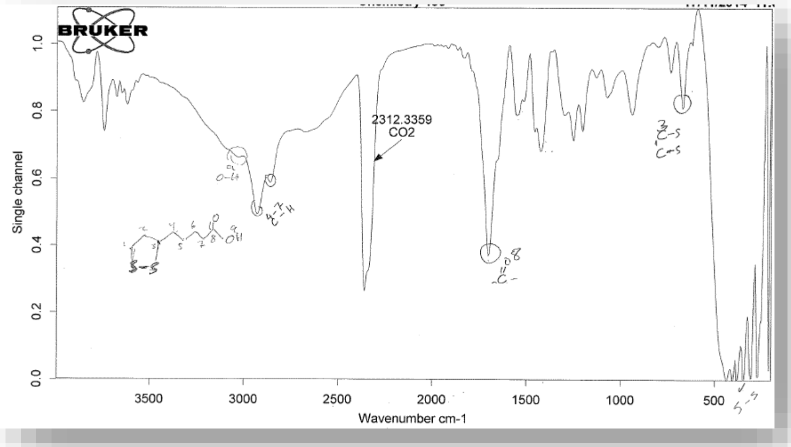

<u>Figure S4. Characterization of Lipoic acid of 1-hydroxy-3- lipo ester (LA-PRO-OH) LA1</u>

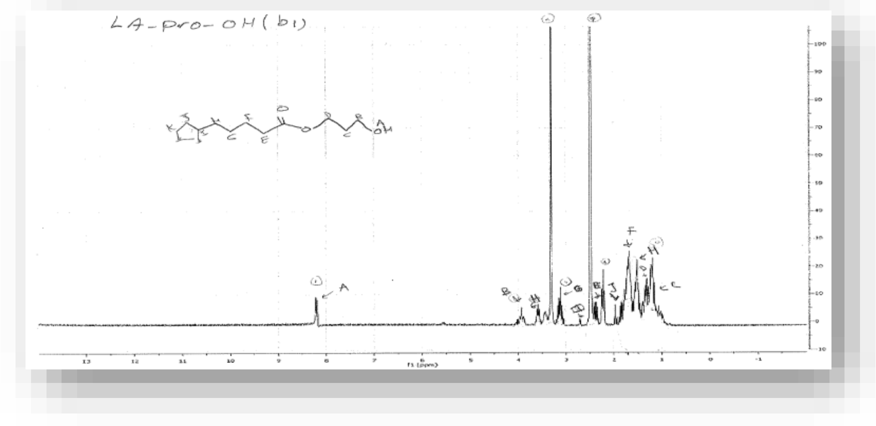

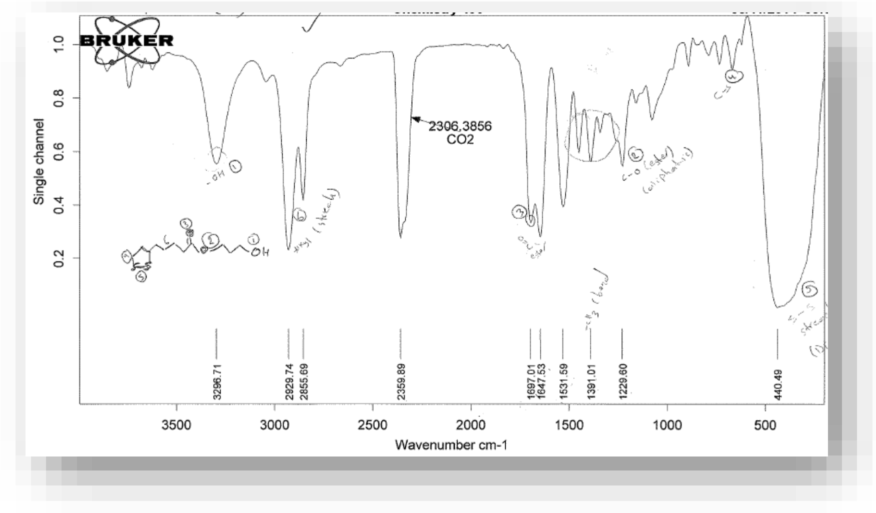

Figure S5. Characterization of 1-hydroxy-6- lipoester (LA-HEX-OH) LA4

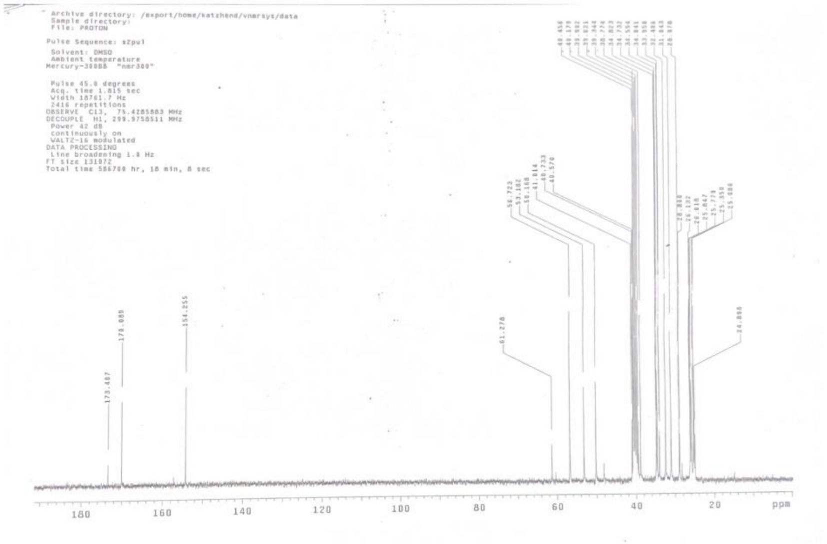

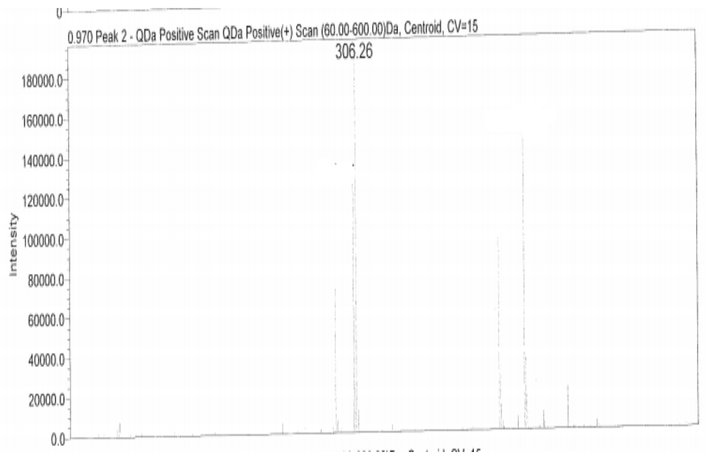

Figure S6. Characterization of Nitrooxy–propane-3-lipoyl ester (LA-PRO-ONO2) LA2.

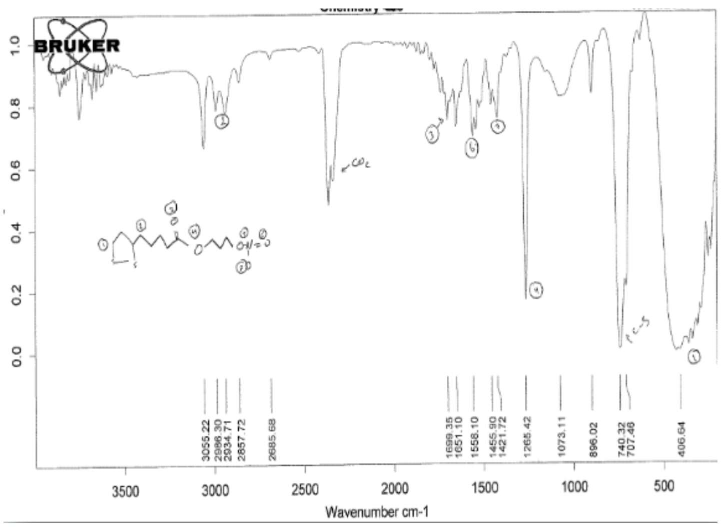

Figure S7. Characterization of 1-Nitrooxy–Hexane-6-lipoyl ester (LA-HEX-ONO2) LA3.

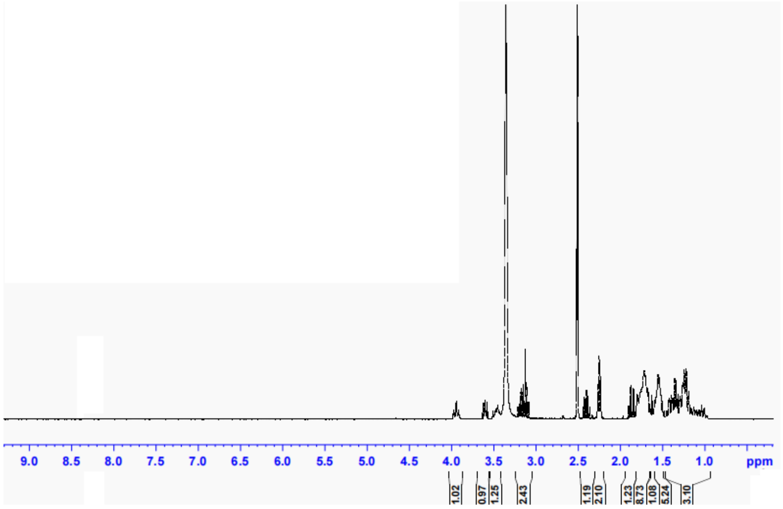

1H NMR (DMSO-d6) δ ppm spectrum

Figure S8. Effect of dichloromethane on GluA1 and GluA2 subunits. Graphs a-d demonstrates the effects of dichloromethane on GluA1 and GluA2 whole-cell current, as well as the rates of deactivation and desensitization. Cells were treated with 12 µM dichloromethane at a concentration comparable to that of the LA derivatives.

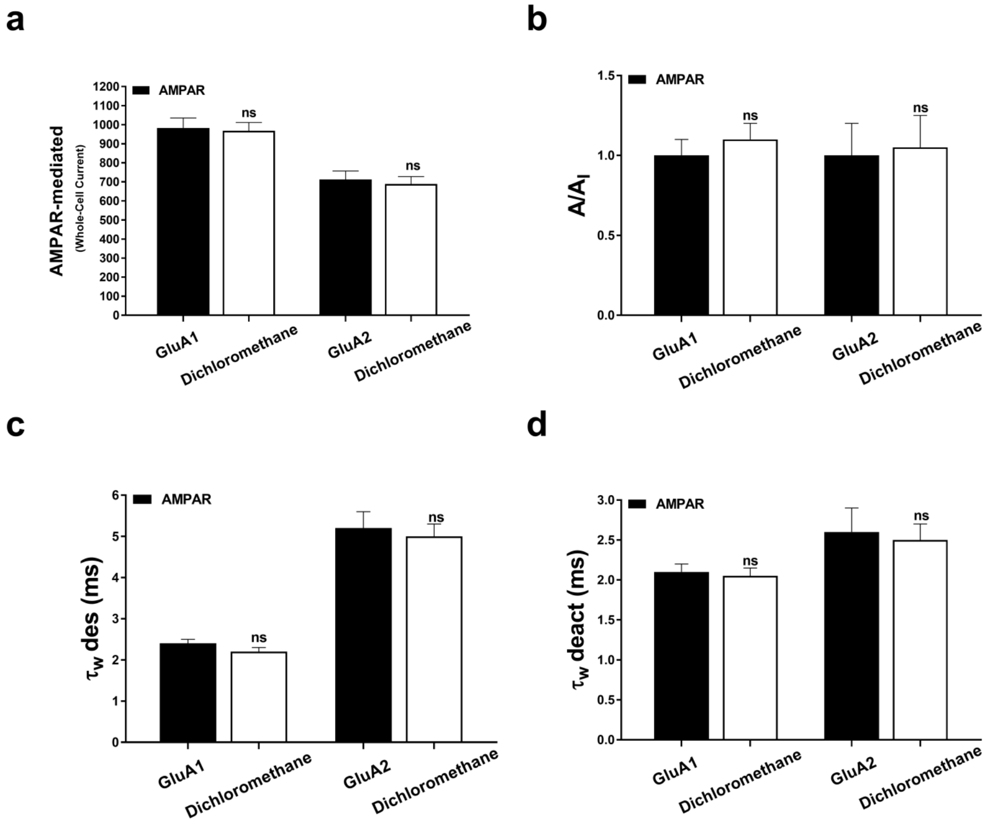
a
AMPAR-mediated
(Whole-Cell Current)
AMPAR
ns
GluA1
Dichloromethane
GluA2
Dichloromethane
b
A/A$_I$
AMPAR
ns
GluA1
Dichloromethane
GluA2
Dichloromethane
c
τ_w des (ms)
AMPAR
ns
GluA1
Dichloromethane
GluA2
Dichloromethane
d
τ_w deact (ms)
AMPAR
ns
GluA1
Dichloromethane
GluA2
Dichloromethane
